# Response Duration During Treatment of Patients with Follicular Lymphoma

**DOI:** 10.3390/biomedicines13061433

**Published:** 2025-06-11

**Authors:** Evelin Kiss, Árpád Illés, Ádám Jóna

**Affiliations:** 1 Department of Hematology, Faculty of Medicine, University of Debrecen, H-4032 Debrecen, Hungary; evlnkiss92@gmail.com (E.K.); illesarpaddr@gmail.com (Á.I.); 2 Doctoral School of Clinical Medicine, University of Debrecen, H-4032 Debrecen, Hungary

**Keywords:** follicular lymphoma, response duration, treatment outcomes, survival analysis, prognostic factors, autologous stem cell transplantation

## Abstract

**Background/Objective**: It is known that the duration of response to treatment in patients with follicular lymphoma decreases with advancing lines of treatment. Our current study investigated progression-free survival in patients with follicular lymphoma undergoing different lines of treatment, aiming to understand the factors influencing treatment response and outcomes. **Methods**: We retrospectively analyzed data from follicular lymphoma patients treated at the University of Debrecen between 2009 and 2023. We collected comprehensive data on patient demographics, disease characteristics, treatment regimens, and outcomes. **Results**: The analysis included 161 patients with follicular lymphoma. The median follow-up for first-line patients was 51 months (range, 3–204), with a median age of 56 years (range, 25–85). The duration of progression-free survival for the first and second lines coincided, while survival in the third line was indeed an ‘unmet medical need’. Factors that influenced the outcome of the first-line treatment of follicular lymphoma patients have been published before by our working group. A univariate analysis of factors that may have influenced the outcome of second-line treatment was performed. A remarkable impact was found in patients undergoing autologous stem cell transplantations and medium-dose treatment. Another important factor was the presence of B-symptoms at the start of second-line treatment. **Conclusions**: Our study confirmed the interesting coincidence of the first- and second-line duration of response in follicular lymphoma patients, which can be explained with the high number of patients undergoing stem cell transplantation and medium-dose treatment in the second line.

## 1. Introduction

Follicular lymphoma (FL) represents the most common subtype of indolent non-Hodgkin lymphoma, characterized by a typically slow-growing nature [1]. While many patients with FL experience prolonged survival, with the introduction of therapies like rituximab pushing median overall survival to nearly 20 years, the disease remains largely incurable with conventional treatments [2]. The management of FL is further complicated by the heterogeneity of its clinical course; although many patients respond well to initial therapies, the majority will ultimately relapse, and the duration of response tends to diminish with each successive line of treatment [3]. Therefore, understanding the factors that influence treatment response and the development of novel strategies to overcome resistance are critical areas of ongoing research [4]. As highlighted by findings detailed in this manuscript, the duration of response to treatment often shortens in patients with follicular lymphoma, underscoring the need for continued investigation into the underlying mechanisms driving this phenomenon and the identification of predictive markers to guide personalized treatment approaches.

## 2. Patients and Methods

A retrospective study was conducted to examine the duration of treatment response in follicular lymphoma patients (grade 3b cases were excluded) treated at the University of Debrecen, Department of Hematology, between February 2009 and September 2023, whose data was available. Histological diagnosis was established according to the World Health Organization classification criteria [5]. Biopsy samples were collected from the most readily accessible site, as histological verification is necessary before PET/CT scanning. FL is classified based on the quantity of centroblasts observed under high-power microscopy. Grade 1 FL exhibits fewer than 5 centroblasts per high-power field, while grade 2 displays 5–15 centroblasts, grade 3A has more than 15 centroblasts, and grade 3B is defined by the presence of centroblast sheets. Patient demographics, disease characteristics (stage, histology, presence or absence of B-symptoms (fever, night sweats, and loss of weight > 10%), absolute lymphocyte (Ly) and monocyte (Mo) counts, and lymphocyte/monocyte (Ly/Mo) ratio), treatment regimens, and staging PET/CT SUVmax data were collected. Disease stage was determined using the Ann Arbor and Lugano classification systems [6,7]. The Follicular Lymphoma International Prognostic Index (FLIPI) was calculated for each patient at diagnosis [8].

The study protocol was approved by the Regional and Institutional Research Ethics Committee of the University of Debrecen (DE RKEB/IKEB 5694–2021). All patients provided written informed consent before treatment initiation, in accordance with the Declaration of Helsinki, to allow for the retrospective collection and publication of their data. Patients were treated based on current institutional guidelines. Treatment was initiated for patients who met the Groupe d’Etude des Lymphomes Folliculaires criteria [9].

PET/CT has been a standard imaging modality employed at the University of Debrecen since May 2009, hence the choice in the investigated period. The Deauville five-point scale is a standardized method used to interpret PET/CT imaging findings in lymphoma patients by comparing the fluorodeoxyglucose uptake in lesions to the activity observed in the liver and mediastinum [6,10]. The progression-free survival (PFS) period was defined as the duration from the initiation of the current line of treatment to the first instance of disease progression, relapse, transformation, or any-cause death [11].

The PET/CT imaging protocol was standardized at the institution. Patients were required to fast for 6 h prior to the procedure, and their blood glucose level was verified upon arrival, with the test only performed if the level was below 12.5 mmol/L. Patient anthropometric data, including weight and height, were recorded. Radiopharmaceuticals were administered using a dedicated injector, Intego, with the dose calculation based on the patient’s body weight. Patients were instructed to rest for 60 min, refraining from activities like watching television or listening to music. They were also advised to drink 1 L of water before and during the waiting time, and to empty their bladders before the scan. The PET/CT acquisition process involved a CT localizer, whole-body CT, and PET imaging, following specific parameters. The PET/CT scans were interpreted by experienced nuclear medicine specialists who were blinded to the clinical data.

The maximum standardized uptake value was measured using the Interview Fusion software, which has cross-validation with the Philips ISP system. The SUVmax was calculated based on the patient’s body weight, with the SUV units expressed in g/mL. A nuclear medicine specialist identified and measured the lesions on the fused PET/CT images, placing regions of interest on the areas with the most intense radiotracer uptake.

We conducted a univariate analysis to identify factors potentially influencing survival outcomes. Continuous variables were categorized, either by exceeding predetermined thresholds or through data-driven cutoff points determined using receiver operating characteristic curve analysis. The outcome variables were defined as events associated with progression-free survival. A multivariable Cox regression model with the Enter method was used to calculate the hazard ratio. Survival analysis was performed using the Kaplan–Meier method, and the log-rank test was employed to compare survival curves. The threshold for statistical significance was set at *p* < 0.05.

## 3. Results

In first-line treatment, 161 patients with follicular lymphoma were analyzed. The median follow-up was 51 months (range, 3–204), and the median age was 56 years (range, 25–85). The patient group included a balanced gender distribution, with a slight majority of females. Over a third of patients presented B-symptoms, and approximately a third had bone marrow involvement. The distribution of follicular lymphoma grades varied, with the majority being grade 1 or 2, and a smaller proportion were grade 3A. In cases of patients with subgroups not determined (ND) according to the expert pathologist opinion, grade 3b was surely excluded, and cases were not further divided among grades 1–3a. Regarding disease stages, IV was the most prevalent. According to the FLIPI score, 38 patients were low-risk, 43 were intermediate-risk, and 52 were high-risk, with missing data for 28 patients, mostly because of lacking information from the referring institution. POD24 (progression of disease within 24 months) was observed in 28 cases. Initial treatment approaches included watch and wait (12 patients), local irradiation (3 patients), R-CVP (rituximab, cyclophosphamide, vincristine, prednisolone) (14 patients), R-CHOP (cyclophosphamide, doxorubicin, vincristine, prednisolone) (73 patients), rituximab (R)/obinutuzumab (G)-bendamustine (58 patients), rituximab (R) monotherapy (1 patient), and rituximab maintenance (96 patients). All first-line patient characteristics are detailed in Table 1.

**Figure 1:**
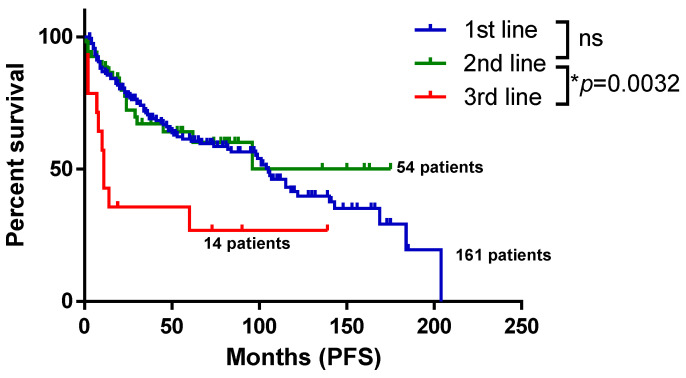
Progression-free survival of follicular lymphoma patients. This figure displays the Kaplan–Meier survival curves illustrating progression-free survival for follicular lymphoma patients in the first, second, and third lines of treatment. PFS—progression-free survival; ns—not significant.

Figure 1 displays the Kaplan–Meier survival curves illustrating PFS for patients in the first, second, and third lines of treatment. The median survival of FL patients treated in the first line was 104 months. As our working group has previously published on factors influencing first-line treatment outcomes in FL patients [2], we do not want to repeat that analysis here. Briefly, we found that SUVmax at staging PET/CT and the Ly/Mo ratio at diagnosis significantly predicted PFS. A combination of staging SUVmax with the Ly/Mo ratio could identify high-risk groups of FL patients.

Median survival for the second line was 96 months. The log-rank test was used to determine the significance of differences between the survival curves of the first and second lines, and the difference was not significant. In contrast, the median survival time for patients in the third line was notably shorter at 11 months. The log-rank test comparing survival curves revealed a significant difference between second line and third line (*p* = 0.0032), showing that third-line treatment was indeed an ‘unmet medical need’.

To look at the difference in response duration in the second and third lines, we analyzed treatment characteristics, which are detailed in Table 2. In the second-line setting, 54 patients were analyzed, with a median follow-up of 30 months. The most common treatment was rituximab (R)/obinutuzumab (G)-DHAP (dexamethasone, cisplatin, cytarabine), administered to 28 patients. Autologous stem cell transplantation (APSCT) was performed in 23 patients. The rest of the treatments included R2 (rituximab + lenalidomide) +/− tafasitamab or epcoritamab as part of clinical trials, rituximab (R)-bendamustine, R-CHOP, and R-CVP. Post-transplant maintenance was administered for nine patients. Other treatment approaches were chosen for six patients (ofatumumab, in toto excisio, local irradiation, rituximab–idelalisib). In the third-line setting, 14 patients were analyzed with a median follow-up of 11 months. Treatments included R-CHOP, R/G-bendamustine, R2, mosunetuzumab, pembrolizumab + vibostolimab combinations as part of a clinical trial, R-zandelisib as part of a clinical trial, duvelisib, R-CHOEP (cyclophosphamide, doxorubicin, vincristine, etoposide) + venetoclax, and R-DHAP.

We conducted a univariate analysis of second-line treatment in follicular lymphoma patients, as detailed Table 3, to explore factors that may influence survival outcomes. These variables include age, treatment, the presence of B-symptoms, tumor grade and stage, bone marrow involvement, bulky disease, hemoglobin levels, lactate dehydrogenase levels, beta-2 microglobulin (B2M) levels, lymphocyte count, monocyte count, the maximum standardized uptake value before the initiation of second-line treatment, undergoing autologous stem cell transplantation, post-transplant maintenance, and elapsed time to transplant from second-line treatment initiation. Although none of the parameters reached statistical significance, there was a trend towards significance with the presence of B-symptoms. Because of the low number of patients in the third line, we did not further investigate it.

**Figure 2:**
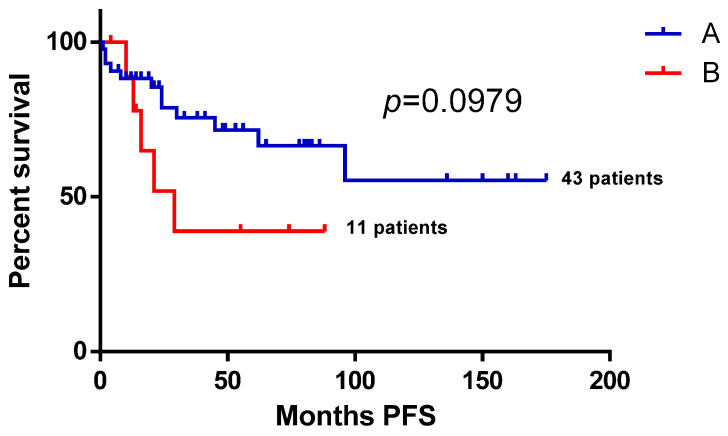
Second-line progression-free survival of follicular lymphoma patients according to B-symptoms. The Kaplan–Meier survival curve visually demonstrates progression-free survival benefit for follicular lymphoma patients without B-symptoms (A) over those who did develop B-symptoms before the initiation of second-line treatment. PFS—progression-free survival.

A Kaplan–Meier survival curve visually demonstrates the previous finding in Figure 2, showing a PFS benefit for those without B-symptoms (A) over those who did develop B-symptoms before the initiation of second-line treatment, while not reaching statistical significance (*p* = 0.0979) with the log-rank test.

**Figure 3:**
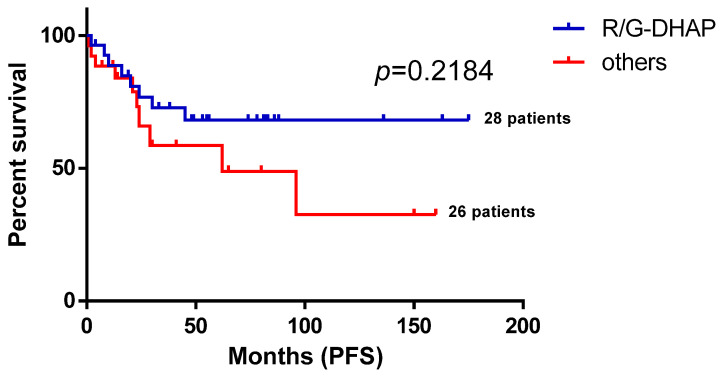
Second-line progression-free survival of follicular lymphoma patients according to the type of treatment. This figure demonstrates a progression-free survival comparison of FL patients who received median-dose treatment (R/G-DHAP) and those who did not and received other 2nd-line treatments like R2 combinations, R-bendamustin, R-CVP, R-CHOP, and other treatment approaches such as ofatumumab, in toto excisio, local irradiation, and rituximab–idelalisib. R—rituximab; G—Obinutuzumab; DHAP—dexamethasone, high-dose cytarabine, and cisplatin; PFS—progression-free survival.

**Figure 4:**
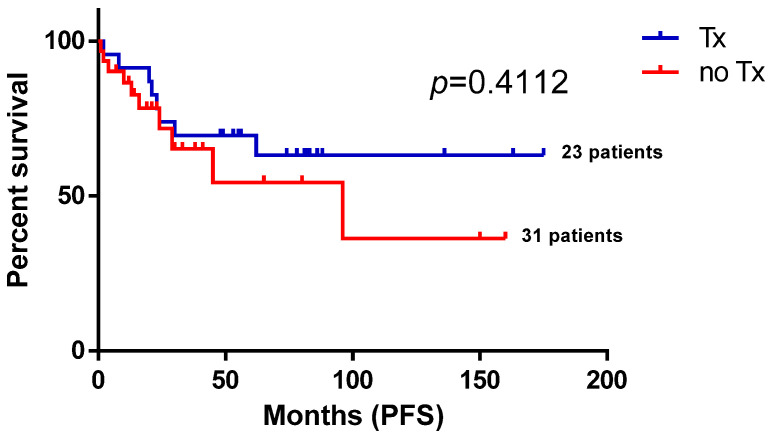
Second-line progression-free survival of follicular lymphoma patients according to autologous peripheral stem cell transplantation. This figure demonstrates a progression-free survival comparison of follicular lymphoma patients who underwent autologous stem cell transplantation and those who did not. Tx—transplantation; PFS—progression-free survival.

Given the high number of patients treated with median-dose treatment (R/G-DHAP) and patients undergoing APSCT in the second line, we decided to explore the hypothesis of whether these treatment approaches contributed to the observed relatively long PFS duration in the second line. As shown in Figure 3, FL patients receiving R/G-DHAP in the second line and who underwent ASCT (Figure 4) experienced numerically improved PFS compared to those who did not, despite not reaching statistical significance (*p* = 0.2184 at R/G-DHAP vs. other second-line treatments (R2 combinations, R-bendamustin, R-CVP, R-CHOP, other treatment approaches (ofatumumab, in toto excisio, local irradiation, rituximab–idelalisib)) and *p* = 0.4112 at APSCT (Tx) vs. no APSCT approach).

The patient number in the third line was very small; therefore, univariate analysis was not performed.

## 4. Discussion

Our study reveals that the PFS durations for first- and second-line treatments in our cohort of follicular lymphoma patients are comparable, while the PFS for third-line treatment is significantly shorter. This underscores the increasing challenge of managing FL as patients progress through subsequent lines of therapy.

One possible explanation for the relatively long PFS observed in the second-line and concurrence with the first-line PFS could be the high proportion of patients undergoing APSCT and receiving medium-dose treatment regimens like R/G-DHAP. Our data suggests a trend towards improved PFS in patients receiving R/G-DHAP followed by APSCT, although this does not reach statistical significance, possibly due to the limited sample size. Further investigation into the impact of these treatment strategies is warranted.

These findings align with previous research demonstrating that response duration (RD) and survival tend to shorten with each relapse in follicular lymphoma. Rivas-Delgado et al. [12] conducted a study on FL patients treated in the rituximab era, demonstrating that response duration and survival shorten after each relapse. The study analyzed 348 newly diagnosed FL patients who received chemoimmunotherapy between 2001 and 2014. After a median follow-up of 6.3 years, the 10-year progression-free and overall survival rates were 53% and 72%, respectively. The 5-year RD for first-, second-, and third-line treatments were 62%, 39%, and 24%, respectively, indicating a significant decrease in RD with each subsequent line of therapy. The study also identified factors predicting longer RD after first-line treatment, including normal beta-2 microglobulin levels, the achievement of complete remission, and maintenance with rituximab. Patients with longer RD after first-line therapy also showed significantly longer RD after second-line therapy.

The SCHOLAR-5 study also highlights the limited data available for patients in the third line of treatment or higher and emphasizes the need for more effective therapies in later lines [13]. The SCHOLAR-5 study was a retrospective cohort study that examined the treatment patterns and outcomes of real-world follicular lymphoma patients in their third line of treatment or higher, a population for whom existing data are limited. The study included adult patients (≥18 years) with grade 1–3a FL, initiating ≥ 3rd line of treatment after June 2014 at major lymphoma centers in the United States of America and Europe. The study found that the likelihood, quality, and duration of clinical response decreases with each subsequent line of therapy, regardless of the type of treatment or geographic region. The study also highlights the need for more effective therapies for patients with relapsed/refractory FL who require therapy beyond the second line.

However, our findings contrast somewhat with the National LymphoCare Study (NLCS) [14], which reported a relatively low fraction of patients receiving multiple therapies during the first several years of management. In our cohort, a significant proportion of patients received second-line treatment, potentially contributing to the observed PFS duration. Also, the NLCS observed that rituximab was frequently utilized in second-line and later treatments for follicular lymphoma, even when patients had previously shown refractoriness to rituximab-based therapy. The NLCS was a prospective cohort study that examined disease characteristics, treatment patterns, and clinical outcomes in a consecutive series of patients diagnosed with follicular lymphoma between March 2004 and March 2007 at participating sites across the United States. The study collected quarterly data on patients’ disease features, subsequent therapies administered, and corresponding treatment responses. The median time from initiation of first-line to second-line treatment was 16 months. Rituximab continued to be utilized in a substantial proportion (70%) of second-line treatments for these follicular lymphoma patients.

Similarly to the previous researchers, Batlevi et al. [15] analyzed over a thousand FL patients diagnosed between 2000 and 2015. While median overall survival (OS) after first-line treatment was not reached, median OS and PFS after second-line treatment were 11.7 years and 1.5 years, respectively. For third-line treatment, median OS was 8.8 years and PFS was 1.1 years. The study found that after fourth-line therapy and beyond, PFS was less than 1 year.

An observational study [16] by the Lymphoma Epidemiology of Outcomes Consortium examined treatment patterns and outcomes for relapsed/refractory FL patients receiving three or more lines of systemic therapy. The median PFS from index therapy (third line or later) was 17 months, with a 2-year PFS of 40%. The 5-year OS from index therapy was 75%. Despite high response rates, median PFS was under 2 years for all therapy classes, with 2-year PFS ranging from 25% to 27% for phosphatidylinositol 3-kinase inhibitor and lenalidomide-based therapy to 48% for salvage and/or cellular therapies. This study provides a benchmark for comparing findings from FL clinical trials.

Several limitations should be considered when interpreting our results. The sample size, particularly in the third-line setting, is relatively small, which may limit the statistical power to detect significant differences. Additionally, the retrospective nature of this study and the fact that these FL patients were treated in a tertiary center introduces the potential for selection bias and confounding factors.

## 5. Conclusions

Despite these limitations, we believe that our study provides valuable insights into the treatment patterns and outcomes of follicular lymphoma patients in the rituximab era. The observation that PFS duration shortens in later lines of therapy highlights the need for novel treatment strategies to overcome treatment resistance and improve long-term outcomes for patients with relapsed/refractory FL. We emphasize the use of median-dose treatment like R/G-DHAP and APSCT as it may prolong second-line PFS. This is in alignment with our previous publication [17], in which we analyzed data from 49 FL patients who received APSCT between 2004 and 2021 and found that age influenced overall survival, while both age and the lymphocyte/monocyte ratio affected PFS. In this study we observed a plateau phase of the curve and a PFS of >98 months, which indicated those considered long-time survivors and “functionally cured”. However, further prospective studies are required to confirm our assumption.

Future research should focus on identifying predictive biomarkers and developing personalized treatment approaches that can effectively address the challenges of managing this heterogeneous disease.

## Figures and Tables

**Table 1:** Characteristics of follicular lymphoma patients treated in first line treatment.

First-Line Treatment			Patient Number
Number of patients			161
Median follow-up (months, range)			51 (3–204)
Median age (years, range)			56 (25–85)
Male/female			69/92
B-symptoms			59
Bone marrow involvement			54
Grade	1		53
	2		55
	3a		34
	ND		19
Stage	I		14
	II		19
	III		51
	IV		77
FLIPI	low		38
	intermediate		43
	high		52
	missing		28
POD24			28
Treatment			
	W&W		12
	Local irradiation		3
	R-CVP		14
	R-CHOP		73
	R/G-bendamustin		58
	R mono		1
	R maintenance		96

ND—not defined; POD24—progression of disease within 24 months; FLIPI—Follicular Lymphoma International Prognostic Index; W&W—watch and wait; R-CVP—rituximab, cyclophosphamide, vincristine, prednisolone; R-CHOP—rituximab, cyclophosphamide, doxorubicin, vincristine, prednisolone; R—rituximab; G—obinutuzumab.

**Table 2:** Patient characteristics of follicular lymphoma patients treated in further lines.

Second-Line Treatment			Patient Number
Number of patients.			54
Median follow-up (months, range)			30 (1–175)
Treatment			
	R/G- DHAP		28
	R2 +/− tafasitamab/epcoritamab		7
	R-bendamustin		3
	R-CHOP		7
	R-CVP		3
	Other		6
	APSCT		23
	PostTx maintenance		9
**Third-line treatment**			
Number of patients			14
Median follow-up (months, range)			11 (2–139)
Treatment			
	R-CHOP		1
	R/G-bendamustin		5
	R2		1
	Mosunetuzumab		1
	Pembolizumab + vibostolimab		1
	R-zandelisib		1
	Duvelisib		1
	R-CHOEP + venetoclax		1
	R-DHAP		2

R—rituximab; G—obinutuzumab; R2—rituximab and lenalidomide combination; DHAP—dexamethasone, high-dose cytarabine, and cisplatin; R-CHOP—rituximab, cyclophosphamide, doxorubicin, vincristine, and prednisolone; R-CVP—rituximab, cyclophosphamide, vincristine, and prednisolone; APSCT—autologous peripheral stem cell transplantation; Tx—transplantation; CHOEP—cyclophosphamide, doxorubicin, vincristine, etoposide, and prednisone.

**Table 3:** Univariate analysis of factors and progression-free survival in second-line treatment of follicular lymphoma patients.

		95% CI for HR		
	Hazard Ratio (HR)	Lower	Upper	Significance
Age	1.025	0.980	1.072	0.288
Treatment	1.927	0.396	1.927	0.417
**B-symptoms**	2.263	0.793	6.461	0.127
Grade	0.491	0.057	4.244	0.518
Stage	1.295	0.115	14.607	0.835
Bone marrow involvement	1.615	0.322	8.083	0.560
Bulky	0.044	0.000	7749.354	0.613
Hgb	0.980	0.941	1.020	0.325
LDH	1.004	0.994	1.013	0.463
B2M	0.929	0.254	3.405	0.912
Ly	0.859	0.265	2.782	0.799
Mo	0.963	0.080	11.662	0.976
SUVmax	1.002	0.932	1.077	0.958
APSCT	0.675	0.262	1.737	0.415
PostTx maintenance	1.799	0.411	7.872	0.436
Time to Tx	1.004	0.980	1.029	0.725

Hgb—hemoglobin; LDH—lactate dehydrogenase; B2M—beta 2 microglobulin; Ly—absolute lymphocyte count; Mo—absolute monocyte count; SUVmax—maximum standardized uptake value; APSCT—autologous peripheral stem cell transplantation; Tx—transplantation.

## Data Availability

Anonymized raw data is available by request.
